# Building the Structure of Our Lives

**Published:** 1922-06

**Authors:** George Wood Clapp


### BUILDING THE STRUCTURE OF OUR LIVES

At the entrance of the little park in which I live, are two monuments built of white marble, one on either side of the street. They are tall and square and at first glance both appear as solid as the land upon which they rest. But they present an important difference which has become more noticeable as the years pass. The top or cap of one was properly formed and cemented; it has withstood the heat of summer and the frost of winter for ten years and is to-day practically as sound as when it was erected.

The other monument was apparently as well builded up to the very top but there the work was incomplete or careless. The expansion under the heat of summer and the contraction in the cold of winter, together with the solvent action of water and the splitting action of frost, have little by little worked their way into crevices in the carelessly finished top and have enlarged the openings, and have here and there split a piece away until the top of the monument is now a wreck and the rest of it promises to become such.

As I have watched the action of the elements on these two monuments one sturdy, strong, useful and ornamental, and the other a slowly crumbling ruin, these monuments have come to represent to my mind a picture of the lives of many dentists.

Two young men sufficiently alike in strength of character, intellectual ability, and aspiration, graduate from dental college. They start to build the monuments of their professional lives. One of them builds entirely with professional material, and what he builds pleases the eye. He serves a satisfactory number of people, lives in a comfortable house, wears good clothes and moves in good society. During the years of construction, the edifice is wholly fair.

The other builds with professional materials joined together with the cement of sound economics. For a time, what he builds may seem no more pleasing to others than the edifice which the first one built with professional materials only.

But when the rains descend and the floods come and increasing family and personal expenses, competition, illness, old age and reduced efficiency have beaten upon both monuments long enough, the difference in texture between them becomes very apparent. The monument in which the elements of professional service have been cemented together by sound economic principles withstands the strain and the storm. The sunshine of prosperity and the frosts of adversity do not split it apart. There may never have been luxury but there is likely always to be comfort.

Under the strains of adversity, the monument built entirely of professional material crumbles like the ruin which I pass daily. Its elements were never linked together properly to withstand adversity in the present or reduction of earnings in old age. And after a few years of apparent success, and pleasing appearance, it goes down in partial or complete failure.

For many years those of us in the profession have been taught to build the monuments of our lives wholly with professional materials. One needs only to look beneath the surface of professional lives about him to see that this advice never was good and that it is apparently successful only through the earlier years of one's life. It can readily be perceived that for the average man such a course merely insures failure in old age if not before, and that in a great number of cases it has insured serious failure in every form of service to one's patients and one's self during middle life.

As I look at the other of the two stone monuments, so strong and secure, bright in the summer's sunshine or piled high with the winter's snows, I am glad that a new perception is dawning in the minds of those of us in the profession which will enable us to build our lives more solidly and securely; that we are learning that the elements of service make a better monument when joined together by the cement of sound economics; that really economic service is cheaper for the patient in the present and more profitable for us in the end; and that we shall best advance the welfare of our profession not by making of our lives monuments which crumble at a touch, but monuments which stand as well against adversity as in prosperity and as honorably in old age as in middle life. —George Wood Clapp in *Digest*.

